# Phylogenetic characterization of the canine distemper virus isolated from veterinary clinics in the Mekong Delta, Vietnam

**DOI:** 10.14202/vetworld.2023.1092-1097

**Published:** 2023-05-24

**Authors:** Tien My Van, Trung Quang Le, Bich Ngoc Tran

**Affiliations:** Faculty of Veterinary Medicine, College of Agriculture, Can Tho University, Can Tho 94000, Vietnam

**Keywords:** canine, distemper virus, hemagglutinin gene, phylogenetic analysis, Vietnam

## Abstract

**Background and Aim:**

Canine distemper (CD) caused by the CD virus (CDV) has a high mortality rate that severely affects dog populations and other terrestrial carnivores worldwide. However, the genetics of CDV strains circulating in various regions in Vietnam, especially the Mekong Delta, remains unclear. This study aimed to characterize the molecular status of CDV strains circulating in the Mekong Delta, Vietnam.

**Materials and Methods:**

Ocular/nasal swabs were collected from 550 dogs with clinically suspected CDV infection from veterinary clinics in three Vietnamese provinces. A reverse transcription-polymerase chain reaction on the part of the hemagglutinin gene was performed. A phylogenetic tree was constructed to analyze the relationship between the detected CDV and GenBank sequences.

**Results:**

The molecular study demonstrated that 4.18% (23/550) of the dogs were positive for CDV. The clinical findings revealed that the positive dogs exhibited clinical signs of distemper. The phylogenetic analysis indicated that the identified CDV sequences were clustered in the same branch with the genotype Asia-1 and distantly related to the vaccine strains. Notably, the CDV sequences detected in this study were grouped with the sequences previously found in southeast Vietnam; however, they were distant from those found in the north.

**Conclusion:**

The present study confirmed the presence of CDV and to the best of our knowledge, highlighted for the first time that the CDV strains circulating in the Mekong Delta of Vietnam belong to the genotype Asia-1.

## Introduction

Canine distemper (CD) is a highly contagious viral disease that seriously affects domestic dogs and wildlife and is associated with high morbidity and mortality worldwide. CD virus (CDV) is a single-stranded RNA virus belonging to the family *Paramyxoviridae* and genus *Morbillivirus* [1, 2]. The complete viral genome contains 15,690 nucleotides, which encode six structural proteins, namely, nucleocapsid, phosphor (P), large (L), matrix, hemagglutinin (H), and fusion (F), and two non-structural proteins (C and V) [1, 2]. The glycoprotein H has largely been acknowledged as an important surface protein of the virus and it plays a key role in adsorption and fusion between the virion and the host cell [1, 3, 4]. Due to its role, the H gene has been widely used in phylogenetic studies of CDV in numerous geographical regions [5–9].

CD is a disease with a worldwide distribution caused by several geographically distinct strains. The first detected strains of CDV were America-1 (including the majority of vaccine strains), America-2, Asia-1, Asia-2, Europe-1/South America-1, and Arctic. Furthermore, the strains in European wildlife, South America-2, Rockborn-like, Africa-1, South America-3, Asia-3, and Asia-4 have been subsequently isolated [6, 10–12]. The circulation of these CDV strains differs among regions and countries [5, 8, 10, 12, 13]. Notably, the new Asia-4 strain was first reported from domestic dogs in Thailand [14], whose adjacency to Vietnam indicates that it shares a similar climate. This raises concerns for other nearby regions regarding the possibility of a change in CDV strains. Thus, there is a need to continuously investigate CDV strains circulating in other geographical regions.

There have been few studies regarding CDV in Vietnam, and the molecular characteristics of CDV in the country were first documented in domestic dogs in 2009 [15] and further described in 2016 [7]. However, the studies performed to date have been limited to only certain parts of the country, and to the best of our knowledge, there have been no studies regarding CD in the Mekong Delta. Thus, it is important to illuminate the molecular characteristics of CDV strains currently circulating in Vietnam, especially in the Mekong Delta, a region with a long history of raising dogs.

This study aimed to determine the phylogenetic characteristics of CDV among dogs clinically suspected of having this infection at veterinary clinics in the Mekong Delta, Vietnam. Our results can help deepen our understanding of the genetic variation of CDV in the investigated region and provide supplementary data for the whole country.

## Materials and Methods

### Ethical approval and informed consent

This study followed Vietnam’s Animal Husbandry Law (32/2018/QH14). All the samples in this study were collected as a part of a diagnostic routine process in veterinary clinics. The signed consent form was obtained from the client-owners for the participation of their dogs in this study.

### Study period and location

The study was conducted from February to October 2022, in three provinces of the Mekong Delta, namely, An Giang, Can Tho, and Tra Vinh. The Mekong Delta is located in the south of Vietnam and is characterized by a tropical monsoon climate. It features two main seasons: the rainy season from May to November and the dry season from December to April. The average annual rainfall is 1500–2500 mm and the average annual temperature is ~27°C [16]. The study area was selected according to the geographical characteristics of the entire region from the coastal zone (Tra Vinh), central zone (Can Tho), and border zone (An Giang). It showed the population characteristic of the entire region from rural to urban. Hence, the selected provinces showed the typical characteristic of rural canine populations (Tra Vinh and An Giang) and urban canine populations (Can Tho) in the Mekong Delta.

### Clinical diagnosis and sample collection

**Figure-1:**
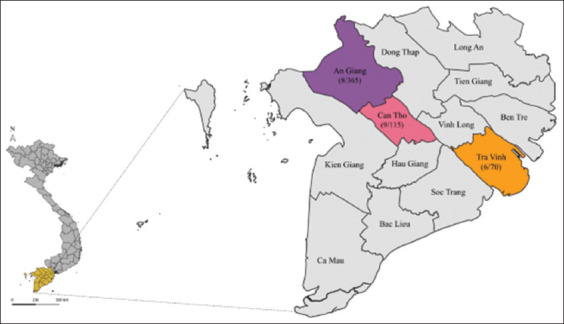
The canine distemper virus samples were collected from An Giang, Can Tho, and Tra Vinh provinces in the Mekong Delta of Vietnam. The letters indicate the name of provinces, the chosen provinces are shown in different colors on the map. The number in the parentheses details the number of positive dogs per number of investigated dogs in each province [Source: The map was constructed using the QGIS software package version 3.22.6].

Ocular/nasal swabs were obtained from 550 dogs with clinical symptoms of CDV infection from private veterinary clinics in three provinces of the Mekong Delta, Vietnam. The number of dogs investigated in each province is presented in Figure-1. A suspicion of CDV was based on the history and typical clinical manifestations of respiratory tract infection, gastroenteritis, and neurological symptom (convulsion, myoclonus). Detailed information regarding age, breed, sex, vaccination status, and clinical symptoms were carefully recorded. The owners of the dogs were informed of the collection of samples and written informed consent was obtained before the procedure. After collection, the samples were dissolved in an Eppendorf tube containing 0.5 mL of phosphate-buffered saline (pH = 7.4) and stored at -80°C until analysis.

### RNA extraction and reverse transcription

Viral RNA was extracted from the ocular/nasal swabs using the SV Total RNA Isolation kit (Promega, Wisconsin, USA) according to the manufacturer’s instructions. Briefly, 200 μL of the homogenized suspension was incubated with 350 μL of RNA Dilution Buffer at 70°C for 3 min, following which 200 μL of 95% ethanol was added. The samples were washed and centrifuged according to the manufacturer’s recommendations.

In the reverse transcription step, cDNA was synthesized using GoScript™ (Promega, Wisconsin, USA) reverse transcription kit according to the manufacturer’s recommendations. The cDNA was stored at -20°C until used for amplification.

### Hemagglutinin gene amplification, sequencing, and phylogenetic analysis

Part of the H gene of CDV was amplified using a previously reported primer pair (forward [5′- TGGTTCACAAGATGGTATTC-3′] and reverse [5′-CAACACCACTAAATTGGACT-3′]). Specifically, this pair amplified a 613 bp fragment of the H gene [5]. The reverse transcription-polymerase chain reaction (RT-PCR) conditions comprised initial denaturation at 94°C for 5 min, then 30 cycles of denaturation at 94°C for 30 s, annealing at 51°C for 30 s, and extension at 72°C for 30 s. The final extension step was conducted at 72°C for 10 min. The amplified RT-PCR products were visualized using 1.5% agarose gel electrophoresis. The positive amplicons were further purified using a purification kit according to the manufacturer’s instructions. After purification, the samples were sent to a commercial laboratory for sequencing using Sanger sequencing. BioEdit software package version 7.2.5 [17] was applied for alignment of the overlapping sequences. A phylogenetic tree was constructed based on the nucleotide sequences in the MEGA software package version 7.0.26 using the maximum likelihood method of phylogenetic tree construction with 1000 bootstrap replications [18]. The nucleotide sequences of this study were compared with previous references from GenBank according to a prior phylogenetic study [6]. Furthermore, CDV sequences from other parts of Vietnam reported in previous studies were selected from GenBank [7, 15].

## Results

### Detection of CDV and associated clinical findings

The overall prevalence of CDV among dogs in the Mekong Delta of Vietnam was 4.18% (23/550 dogs). The rate of positivity was highest in the Tra Vinh province (8.57%) followed by Can Tho (7.83%) and then An Giang (2.19%). The age of the infected dogs ranged from 2 months to 7 years, and six (26.09%) dogs reportedly underwent complete vaccination. The majority of CDV-positive cases exhibited respiratory symptoms (cough, dyspnea, and purulent nasal discharge) (86.95%). The next most common symptom type was gastrointestinal symptoms, including vomiting and diarrhea, which were observed at a rate of 60.87%. Furthermore, neurological symptoms (convulsion and myoclonus) were occasionally detected at a rate of 21.74% (Table-1).

**Table-1:** The summary of signalments, vaccination status, collected location, and clinical symptoms of 23 CDV-positive dogs.

Case no.	Age	Breed	Sex	Vaccination status	Province	Clinical symptoms
VN-TV01-2022	3 months	Pure	Male	No	Tra Vinh	RS, GS
VN-TV02-2022	10 months	Mixed	Male	No	Tra Vinh	RS, GS, NS
VN-TV03-2022	2 years	Mixed	Female	Yes	Tra Vinh	GS
VN-TV04-2022	6 months	Mixed	Male	No	Tra Vinh	RS, NS
VN-TV05-2022	3 years	Mixed	Female	No	Tra Vinh	RS
VN-TV06-2022	5 years	Mixed	Female	No	Tra Vinh	RS, GS
VN-CT01-2022	6 months	Pure	Male	No	Can Tho	RS, NS
VN-CT02-2022	4 years	Mixed	Female	Yes	Can Tho	GS
VN-CT03-2022	1 year	Pure	Female	Yes	Can Tho	RS
VN-CT04-2022	8 months	Mixed	Female	No	Can Tho	RS, GS, NS
VN-CT05-2022	2 years	Pure	Male	No	Can Tho	RS
VN-CT06-2022	7 years	Mixed	Male	No	Can Tho	RS, GS
VN-CT07-2022	11 months	Pure	Female	No	Can Tho	RS, GS
VN-CT08-2022	4 years	Mixed	Female	No	Can Tho	RS
VN-CT09-2022	3 years	Pure	Male	Yes	Can Tho	GS
VN-AG01-2022	2 years	Mixed	Female	No	An Giang	RS, GS
VN-AG02-2022	1 year	Mixed	Female	Yes	An Giang	RS, GS
VN-AG03-2022	2 months	Pure	Male	No	An Giang	RS, NS
VN-AG04-2022	9 months	Mixed	Male	No	An Giang	RS
VN-AG05-2022	7 months	Mixed	Male	No	An Giang	RS, GS
VN-AG06-2022	1 year	Mixed	Male	Yes	An Giang	RS, GS
VN-AG07-2022	4 years	Mixed	Female	No	An Giang	RS, GS
VN-AG08-2022	6 years	Pure	Female	No	An Giang	RS

RS=Respiratory symptom (cough, dyspnea, and purulent nasal discharge); GS=Gastrointestinal symptom (vomiting and diarrhea); NS=Neurological symptom (convulsion and myoclonus)

### Comparison of the percentage identity between the detected CDV strains and others from GenBank

Herein, 23 sequences of a part of the H gene were determined and compared with the widely found strains specific to particular geographical areas in GenBank. The highest rates of nucleotide identities were detected among the genotype Asia-1, up to 96.67%–97.02%. The nucleotide identities between the CDV sequences of the present study and vaccine strains (America-1 and Rockborn-like) were in the ranges of 91.96%–95.02% and 92.35%–94.87%, respectively. Notably, the percentage identity of the nucleotides among the detected CDV sequences and past CDV sequences in Vietnam was 92.69%–94.59%. Similarly, the percentage of amino acid identity among the CDV sequences of this study and genotype Asia-1 was the highest, reaching 98.66%–99.58%. The amino acid identities between the present CDV sequences and America-1 (vaccine) strains were 96.84%–97.38% and that between the present CDV sequences and the Rockborn-like (vaccine) strains were 96.77%–97.55%, whereas those between the present CDV sequences and the earlier CDV sequences in Vietnam ranged from 96.91% to 97.23% (Table-2).

**Table-2:** Comparison of the nucleotide and amino acid sequence identity values among 23 detected CDV samples in this study and the reference strains from GenBank.

Strain	(%) identity with 23 CDV sequences in this study	

	Nucleotide	Amino acid
America-1	92.35–94.87	96.84–97.38
America-2	92.07–92.69	97.07–97.23
Africa-1	91.96–93.51	96.80–96.99
Africa-2	92.06–93.37	96.80–96.99
Arctic	91.31–94.66	96.86–97.51
Asia-1	96.67–97.02	98.66–99.58
Asia-2	91.68–93.38	96.80–97.19
Asia-3	92.24–93.51	96.80–96.92
Asia-4	90.35–91.98	96.84–96.99
Europe-1/South America-1	91.89–93.56	96.82–97.56
European wildlife	91.91–93.40	96.80–97.00
Rockborn-like	91.96–95.02	96.77–97.55
South America-2	91.94–95.04	96.82–97.44
South America-3	91.93–93.35	96.75–96.92
Other Vietnamese strains	92.69–94.59	96.91–97.23

CDV=Canine distemper virus

### Phylogenetic analysis

**Figure-2:**
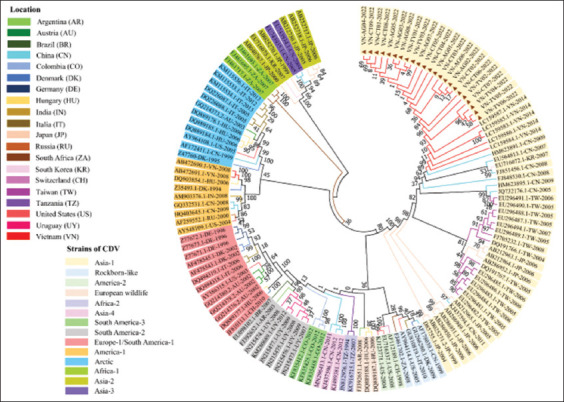
The phylogenetic tree was constructed from the partial hemagglutinin (H) gene sequence of 23 detected canine distemper virus (CDV) samples in the Mekong Delta with a length of 613 bp and 109 full-length H genes of 14 previous strains. The brown triangle indicates the detected CDV sequences in this study. The color of the branches indicates the original country of the individual CDV strains. The strains are arranged by Genbank accession number-country abbreviations-isolated year, the color indicates the genotype of the virus. The tree was constructed through the nucleotide sequences, using the Maximum likelihood method with 1000 bootstrap replications.

The CDV sequences detected from dogs in this study were identical to the CDV sequences isolated from Ho Chi Minh City in Vietnam and located in the same group as others from China, South Korea, Taiwan, and Japan within the Asia-1 genotype. All the CDV variants detected in our study were grouped into the same cluster; however, they were segregated into a subgenotype branch and distantly correlated with the vaccine (America-1 and Rockborn-like) strains and other geographical strains around the world (Figure-2).

## Discussion

Although modified live vaccines against CDV infection are available, CD continues to be identified among dogs in various geographical regions [9, 19–22]. This demonstrates that CD remains an important disease among dog populations worldwide. Notably, new CDV strains have been proposed and confirmed recently [10, 14, 23] due to the high mutation and replication rate of single-stranded RNA viruses [24]. Thus, there is a need for supplementary information on the prevalence and molecular characteristics of the CDV epidemic. At least two CDV strains were reported in Vietnam, namely, America-1 in the north of the country [15] and Asia-1 in the southeast [7]. This prompted us to perform the present study, the results of which indicated for the first time that the CDV sequences detected from dogs in the Mekong Delta of Vietnam belong to the genotype Asia-1.

Herein, 4.18% of the investigated dogs were positive for CDV, as determined through RT-PCR, and all the positive animals exhibited almost typical clinical manifestations of CDV infection. This is consistent with previous observations that most infected dogs demonstrate the clinical characteristics of CD [1–3]. In Vietnam, there have been few epidemiological studies regarding CD in domestic dogs; however, this study showed that dogs aged from 2 months to 7 years old could be infected with CDV. The number of dogs that had undergone complete vaccination was low, which could be related to ancient breeding practices and the economic situation of the owners. This may explain the prevalence of CDV in the investigated region.

There is a need for studies involving the molecular analysis of CDV in the Mekong Delta because few studies have indicated CDV infection in dogs from other regions in Vietnam. Previous studies have analyzed the molecular characteristics of the H and P genes of the CDV strains isolated from domestic dogs [7, 15]. Herein, all the detected CDV sequences based on the part of the H gene were clustered in the same group as others from China, South Korea, Taiwan, and Japan and belonged to the Asia-1 genotype. This finding agrees with that of a previous report describing that there was a correlation between Vietnamese CDV strains and those of the neighboring countries [7]. Notably, our study mentioned the same CDV genotype (Asia-1) in Ho Chi Minh City [7], while the samples were distant from those reported in a previous study in the north of Vietnam, which showed the genotype of America-1 [15]. The current results imply that the prevalence of CDV could be associated with geographical origin rather than host specificity. Similarly, a previous study has revealed that the circulation of CDV strains was similar to that in neighboring geographical regions [25].

Herein, two vaccine strains, namely, America-1 (including the vaccine strains Convac and Snyder Hill) and Rockborn-like, were selected for comparison with the field strains according to a previous phylogenetic study [6]. Remarkably, 6/23 (26.09%) CDV-positive dogs displayed a distant relationship with two vaccine strains, although they had previously been reported with a vaccination history. These results suggest that the cause of CDV-positive dogs may be related to vaccination failure. The vaccine quality and poor immune response of dogs to the vaccine could lead to vaccination failure [26].

### Limitations of the study

The present study had a range of limitations. First, the full-length H gene sequence was not obtained. Instead, in this study, only a part of the H gene in the CDV sequences isolated from dogs in veterinary clinics was sequenced. The advantage of using different genetic targets (H, P, N, and F genes or combining them together) and the different sizes of the target genes have led to diverse datasets for CDV sequences. However, these features have also led to difficulty in performing valid comparisons among studies [5]. Another limitation is that the field vaccine strains circulating in Vietnam were not sequenced in this study. Further studies should be conducted to overcome these limitations to clearly illustrate the relationship between the field strain CDVs and field strain vaccines in Vietnam.

## Conclusion

The CDV sequences detected from 23 clinically suspected dogs in the Mekong Delta of Vietnam were characterized as belonging to the Asia-1 genotype as revealed through phylogenetic analysis of the partial H gene sequences. The data obtained here also provide some epidemiological characteristics associated with the disease. Further larger-scale molecular studies are warranted to determine the CDV strains present throughout the country, and recombination between field CDV strains and vaccine strains should be considered a strategy for controlling the CD epidemic in the future.

## Authors’ Contributions

BNT: Conceptualized and designed the study. TMV and BNT: Collected field samples. TMV, TQL, and BNT: Performed molecular experiments, analyzed, and interpreted the data. TQL and BNT: Wrote the first draft. TMV, TQL, and BNT: Revised the manuscript. All authors have read, reviewed, and approved the final manuscript.
